# Supplementary material for: Current Expertise, Opinions, and Attitude toward TNF-⍺ Antagonist Biosimilars among Physicians: A Self-Administered Online Survey in Western Switzerland
Source: Healthcare (Basel). 2022 Oct 28;10(11):2152. doi: 10.3390/healthcare10112152 (PMC9690245; doi:10.3390/healthcare10112152)
Supplement: Supplementary file 1 [file healthcare-10-02152-s001.zip › healthcare-1954438-supplementary.pdf]

Supplementary Material

TABLE OF CONTENTS

I. SURVEY 2

II. RESULTS AND STATISTICS 4

II.1. PARAMETRIC AND NONPARAMETRIC METHODS USED 4
II.2. CHARACTERISTICS 5
II.2.1. NUMBER OF PARTICIPANTS 5
II.2.2. AGE 5
II.2.3. YEARS OF CUMULATED EXPERIENCE 7
II.3. EXPERTISE AND KNOWLEDGE REGARDING BIOSIMILARS 9
II.3.1. QUESTION 1 9
II.3.2. QUESTION 2 10
II.3.3. QUESTION 3 10
II.3.4. QUESTION 4 14
II.3.5. QUESTION 5 15
II.4. CONFIDENCE IN TNF-α ANTAGONIST BIOSIMILARS 15

I. SURVEY

1. How much do you agree with the following statements? (*strongly disagree, disagree, Neither agree nor disagree, agree, strongly agree*)
    1. “In general, my level of knowledge about biosimilars is good”
    2. “In general, I feel well informed about biosimilars”
    3. “I feel comfortable talking to my patients about the benefits of biosimilars”
    4. “I feel comfortable talking to my colleagues about the benefits of biosimilars”
2. In the past 12 months, how often have you used the following sources of information about biosimilars? (*never, once every 12 months, 1-2 times every 6 months, 1-2 times every 3 months, 1-2 times every month, at least once a week*)
    1. Self-learning and scientific publications
    2. The pharmaceutical industry (including medical representatives)
    3. The website of the Swiss Agency for Therapeutic Products (swissmedic.ch)
    4. The website of the Federal Office of Public Health (FOPH), including the list of specialties (LS) (bag.admin.ch)
    5. The website for information on medicines (swissmedicinfo.ch)
    6. The website of the Swiss Compendium of Medicines (compendium.ch)
    7. Professional congresses
    8. The media
    9. My medical colleagues
    10. My pharmacist colleagues
3. Which statements about biosimilars do you think are correct? (*Check all that apply*)
    1. A biosimilar has an identical amino acid sequence and glycosylation to its reference biologic. (*FALSE*)
    2. A biosimilar is similar to a patent-expired reference biologic. (*TRUE*)
    3. A biosimilar has no differences in therapeutic effectiveness from its reference biologic. (*TRUE*)
    4. A biosimilar has no differences in safety (adverse events and serious adverse events) from its reference biologic. (*TRUE*)
    5. A biosimilar does not require preclinical and clinical studies. (*FALSE*)
    6. A biosimilar has the same immunogenicity as its reference biologic. (*TRUE*)
    7. A biosimilar may have minor structural variations in its amino acid sequence from one production batch to another. (*FALSE*)
4. For which of the following reference biologics (in bold) have you ever prescribed a biosimilar (in italics)? (*Check all that apply*)
    1. None of the above
    2. Amgevita® or Hulio® or Hyrimoz® or Imraldi® or Idacio® → (reference biologic: Humira®)
    3. Benepali® or Erelzi® → (reference biologic: Enbrel®)
    4. Inflectra® or Remsima® → (reference biologic: Remicade®)
5. In the past 12 months, how often did you prescribe or refill a prescription for any of the biosimilars below? (*never, once every 12 months, 1-2 times every 6 months, 1-2 times every 3 months, 1-2 times every month, at least once a week*)
    1. Amgevita® or Hulio® or Hyrimoz® or Imraldi® or Idacio®
    2. Benepali® or Erelzi®
    3. Inflectra® or Remsima
6. How much do you agree with the following statements? (*strongly disagree, disagree, Neither agree nor disagree, agree, strongly agree*)
    1. “I prescribe a biosimilar to a patient based primarily on my clinical experience”
    2. “Using the literature, I gladly prescribe a biosimilar to patients who have not yet started treatment with the reference biologic”
    3. “Using the literature, I gladly discuss with my patients in remission and under treatment with a reference biologic to propose a substitution with the biosimilar”
    4. “I present information about biosimilars in a positive way in discussions with my patients.”
    5. “When a biosimilar comes on the market, I prefer to wait for the results of substitution in my colleagues' patients before proposing the substitution to my patients”
    6. “I prefer to initiate treatment with the biosimilar in patients who have not yet started treatment with the reference biologic, rather than substituting my patients who are already in remission with the reference biologic”
    7. “I refuse to substitute a biosimilar for the reference biologic in a patient who has had difficulty achieving remission”
    8. “I do not prescribe biosimilars because they are not available in my institution”

9. “I explicitly use the term 'biosimilar' when talking to my patients who are going to start a biosimilar or to whom I want to suggest a biosimilar substitution”
10. “My patients who are in remission are willing to substitute their reference biologic with the biosimilar”
11. “Patients who have not yet started treatment with the reference biologic are willing to start treatment with the biosimilar”
12. “I have given up prescribing biosimilars because health insurance companies regularly hinder their reimbursement for off-label use”

7. How much do you agree with the following statements? (*strongly disagree, disagree, Neither agree nor disagree, agree, strongly agree*)
    1. “The prescription of biosimilars is promoted by the Federal Office of Public Health (FOPH)”
    2. “The prescription of biosimilars is promoted by my institution/office”
    3. “Biosimilars are an opportunity to reduce health care costs”
    4. “The lack of incentive systems is a barrier to the prescription of biosimilars in Switzerland”
    5. “I support the routine prescribing of biosimilars in place of the original biologics in patients who have not yet started treatment with the reference biologic.”
    6. “I have enough information about the safety of use (adverse events and serious adverse events) of biosimilars to be comfortable with their prescription”
    7. “I have enough information about the therapeutic efficacy of biosimilars to be comfortable with their prescription”
    8. “Patients who have not yet started treatment with the reference biologic should start treatment with a biosimilar, if one exists”
    9. “I have enough time to offer a biosimilar to my patients who have not yet started treatment with the reference biologic and to explain the rationale for it”
    10. “I have enough time to propose a biosimilar substitution to my patients who are already on a reference biologic and in remission and to explain the rationale for it”
    11. “Prescribing a biosimilar gives me confidence in the therapeutic management of my patients who are already on a reference biologic and in remission”
    12. “Prescribing a biosimilar gives me confidence in the therapeutic management of my patients who have not yet started their treatment with the reference biologic”
    13. “The success of the substitution of a biologic by its biosimilar depends mainly on the physician-patient relationship”
8. What is your age?
9. In which canton do you currently practice your primary specialty? (*Bern, Fribourg, Geneva, Jura, Neuchâtel, Valais, Vaud*)
10. In what setting are you currently practicing (*Check all that apply*)
11. How many years of cumulative active experience do you have in your specialty(ies)
12. Do you practice propharmacy (direct dispensing of medications by the prescribing physician)? (*Yes, No*)
13. What are the major illnesses of the patients you manage? (*Check all that apply*)

II. RESULTS AND STATISTICS

II.1. Parametric and nonparametric methods used

Analysis of variance of the dependent variables was performed using Bartlett's and Levene's tests.

Supplementary Table S1. Summary of the analyses performed on the dependent variables.

Dependent variable	Independent variable	Method	Test	*post hoc* test
Age	Specialty	Parametric	Welch's ANOVA	Tukey
Age	Place of practice	Parametric	Student's *t* test	–
Years of CE	Specialty	Nonparametric	Kruskal-Wallis rank sum test	Pairwise Wilcoxon rank sum test with continuity correction
Years of CE	Place of practice	Nonparametric	Wilcoxon rank sum test	–
KS	Specialty	Parametric	ANOVA	Tukey
KS	Place of practice	Parametric	ANOVA	Tukey
KS	Years of CE	Parametric	Student's t test	–
5 and 6 Point LSQs	Specialty	Nonparametric	Kruskal-Wallis rank sum test	Pairwise Wilcoxon rank sum test with continuity correction
5 and 6 Point LSQs	Years of CE	Nonparametric	Kruskal-Wallis rank sum test	Pairwise Wilcoxon rank sum test with continuity correction
5 and 6 Point LSQs	Place of practice	Nonparametric	Wilcoxon rank sum test	–
5 and 6 Point LSQs	KS	Nonparametric	Likelihood ratio tests of cumulative link models	Parallel regression assumption

ANOVA = Analysis of variance, CE = cumulative experience, KS = knowledge score, LSQs = Likert scale questions

II.2. Characteristics

II.2.1. Number of participants

Supplementary Table S2. Total number of participants per specialty. Percentages have been rounded and are for information purposes only. Totals do not necessarily add up to 100%.

Specialty	number (%)
GAS	4 (11 %)
IMM	11 (30 %)
RHE	22 (59 %)
Total	37 (100 %)

GAS = gastroenterology, IMM = immunoallergology, RHE = rheumatology

Supplementary Table S3. Total number of participants per place of practice. Percentages have been rounded and are for information purposes only. Totals do not necessarily add up to 100%.

Place of practice	number (%)
Office	7 (19 %)
Hospital	30 (81 %)

Supplementary Table S4. Total number of participants per years of cumulative experience. Percentages have been rounded and are for information purposes only. Totals do not necessarily add up to 100%.

	number (%)
< 10 years	18 (49 %)
Between 10 and 20 years	8 (22 %)
> 20 years	11 (30 %)

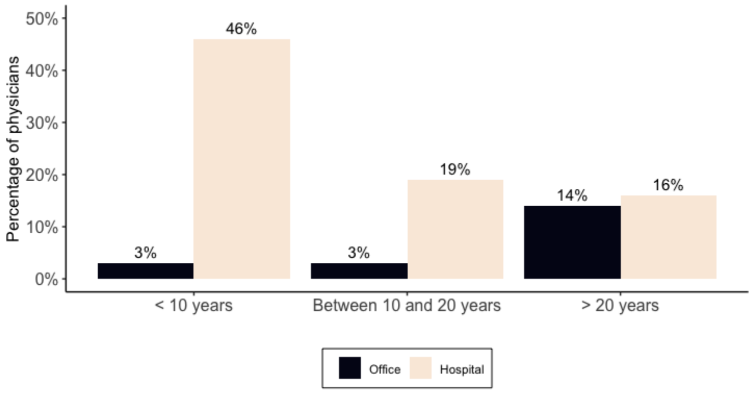

Supplementary Figure S1. Percentages of physicians per years of cumulative experience and place of practice. Percentages have been rounded and are for information purposes only. Totals do not necessarily add up to 100%.

II.2.2. Age

By specialty

Supplementary Table S5. Mean age, by specialty. Percentages have been rounded and are for information purposes only. Totals do not necessarily add up to 100%.

Specialty	number	mean (sd)
GAS	4	36 (3)
IMM	11	36 (5)
RHE	22	50 (12)
Total	37	44 (12)

GAS = gastroenterology, IMM = immunoallergology, RHE = rheumatology

Supplementary Table S6. Analysis of variance of age, by specialty

Test	p-value
Bartlett	1.60E-03
Levene	1.34E-03

Analysis of mean age

→ One-way analysis of means (not assuming equal variances):

- F statistic = 9.4594,
- p-value = 2.80E-03

Pairwise analysis

→ Tukey's WSD post-hoc test of means for unequal variance and sample size

Supplementary Table S7. Results from the Tukey post-hoc test

Groups	q statistic	p value
GAS vs IMM	0.32	0.97
GAS vs RHE	6.79	4.29E-04
IMM vs RHE	6.93	8.75E-05

GAS = gastroenterology, IMM = immunoallergology, RHE = rheumatology

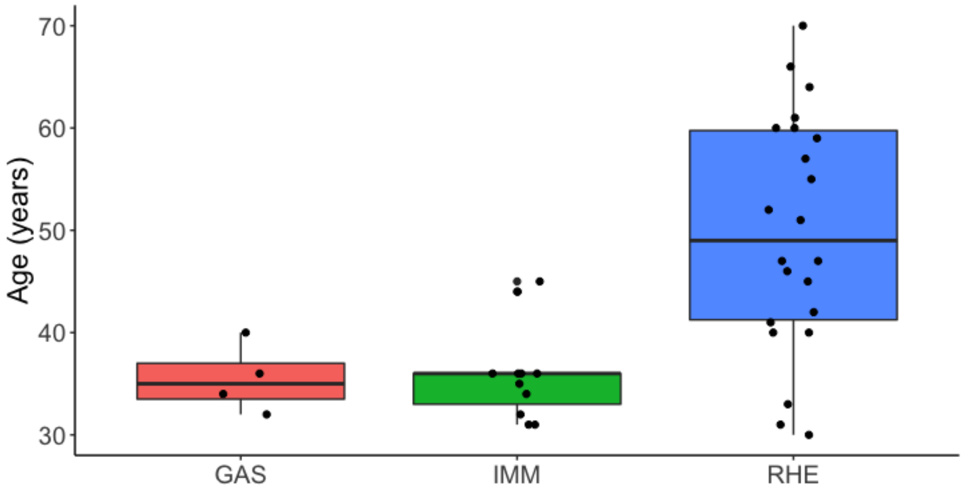

Supplementary Figure S2. Box plot of participants' age, by specialty. *GAS = gastroenterology, IMM = immunoallergology, RHE = rheumatology*

By place of practice

Supplementary Table S8. Mean age, by place of practice

Place of practice	number	mean (sd)
Office	7	58 (7)
Hospital	30	41 (10)
Total	37	44 (12)

Supplementary Table S9. Analysis of variance of age, by place of practice

Test	p-value
Bartlett	0.38
Levene	0.44

Analysis of mean age

→ Two Sample *t*-test :

- t statistic = 4.218,
- p-value = 1.65E-04

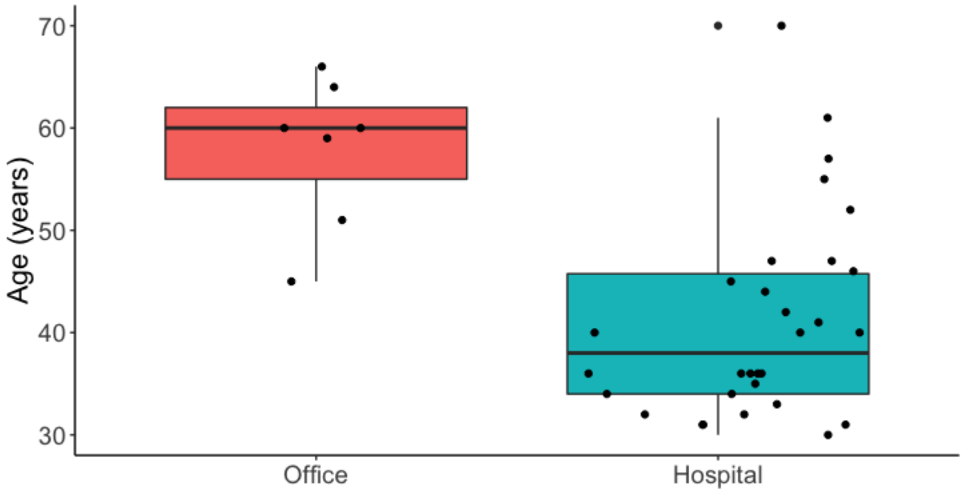

Supplementary Figure S3. Box plot of participants' age, by specialty. *GAS = gastroenterology, IMM = immunoallergology, RHE = rheumatology*

II.2.3. Years of cumulated experience

By specialty

Supplementary Table S10. Number of participants per years of cumulated experience and specialty. Percentages have been rounded and are for information purposes only. Totals do not necessarily add up to 100%.

Specialty	< 10 years *n* (%)	Between 10 and 20 years *n* (%)	> 20 years *n* (%)
GAS	3 (8 %)	1 (3 %)	—
IMM	9 (24 %)	1 (3 %)	1 (3 %)
RHE	6 (16 %)	6 (16 %)	10 (27 %)
Total	18 (49 %)	8 (22 %)	11 (30 %)

GAS = gastroenterology, IMM = immunoallergology, RHE = rheumatology

Analysis of ordinal data

→ Kruskal-Wallis rank sum test:

- chi-squared = 9.9582,
- p-value = 6.88E-03

Pairwise analysis

→ Wilcoxon rank sum test

Supplementary Table S11. Results from the Tukey post-hoc test.

p-values	GAS	IMM
IMM	0.93	-
RHE	0.08	0.02

GAS = gastroenterology, IMM = immunoallergology, RHE = rheumatology

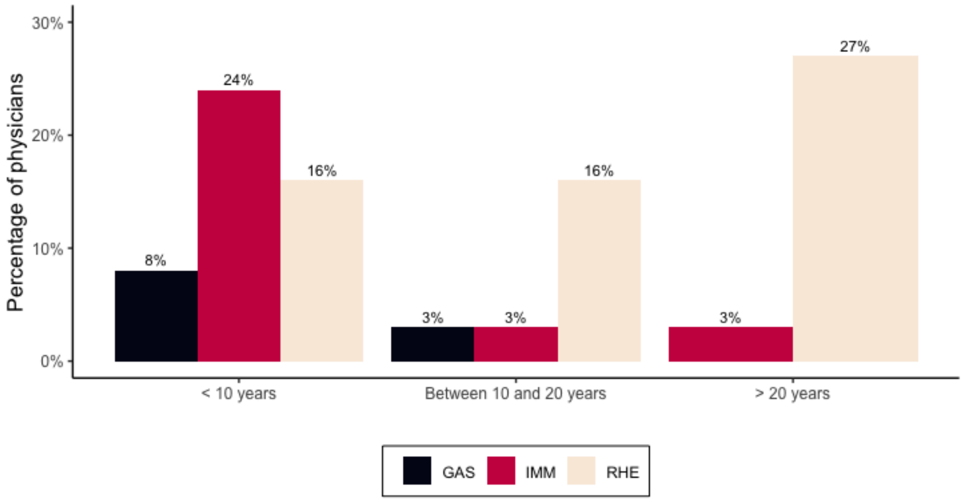

Supplementary Figure S4. Percentages of patients per years of cumulative experience and specialty. Percentages have been rounded and are for information purposes only. Totals do not necessarily add up to 100%. *GAS = gastroenterology, IMM = immunoallergology, RHE = rheumatology*

By place of practice

Supplementary Table S12. Number of participants per years of cumulated experience and place of practice. Percentages have been rounded and are for information purposes only. Totals do not necessarily add up to 100%.

	**< 10 years *n* (%)**	**Between 10 and 20 years *n* (%)**	**> 20 years *n* (%)**
Office	1 (3 %)	1 (3 %)	5 (14 %)
Hospital	17 (46 %)	7 (19 %)	6 (16 %)
Total	18 (49 %)	8 (22 %)	11 (30 %)

Analysis of ordinal data

→ Wilcoxon rank sum test:

- W = 188.5,
- p-value = 1.26E-03

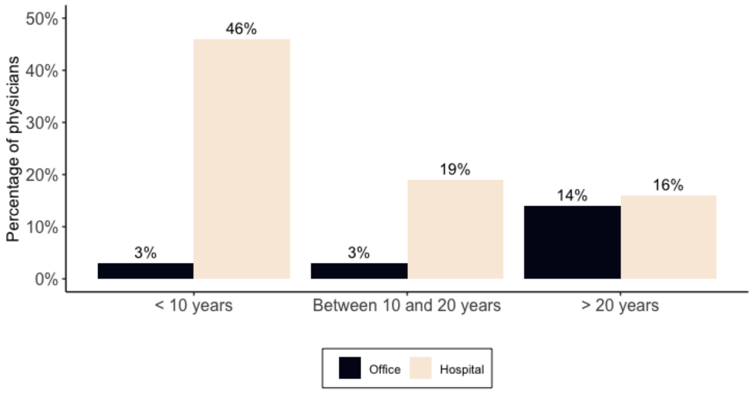

Supplementary Figure S5. Percentages of patients per years of cumulative experience and place of practice. Percentages have been rounded and are for information purposes only. Totals do not necessarily add up to 100%.

II.3. Expertise and knowledge regarding biosimilars

II.3.1. Question 1

Q1: How much do you agree with the following statements?
q1.1 - “In general, my level of knowledge about biosimilars is good”
q1.2 - “In general, I feel well informed about biosimilars”
q1.3 - “I feel comfortable talking to my patients about the benefits of biosimilars”
q1.4 - “I feel comfortable talking to my colleagues about the benefits of biosimilars”

Supplementary Table S13. Ordinal data for questions q1.1-4. Percentages have been rounded and are for information purposes only. Totals do not necessarily add up to 100%.

	**q1.1** ***n*** **(%)**	**q1.2** ***n*** **(%)**	**q1.3** ***n*** **(%)**	**q1.4** ***n*** **(%)**
Strongly disagree	0	1 (3 %)	1 (3 %)	1 (3 %)
Disagree	5 (14 %)	6 (16 %)	4 (11 %)	4 (11 %)
Neither disagree nor agree	7 (19 %)	4 (11 %)	2 (5 %)	1 (3 %)
Agree	16 (43 %)	14 (38 %)	16 (43 %)	13 (35 %)
Strongly agree	9 (24 %)	12 (32 %)	14 (38 %)	18 (49 %)

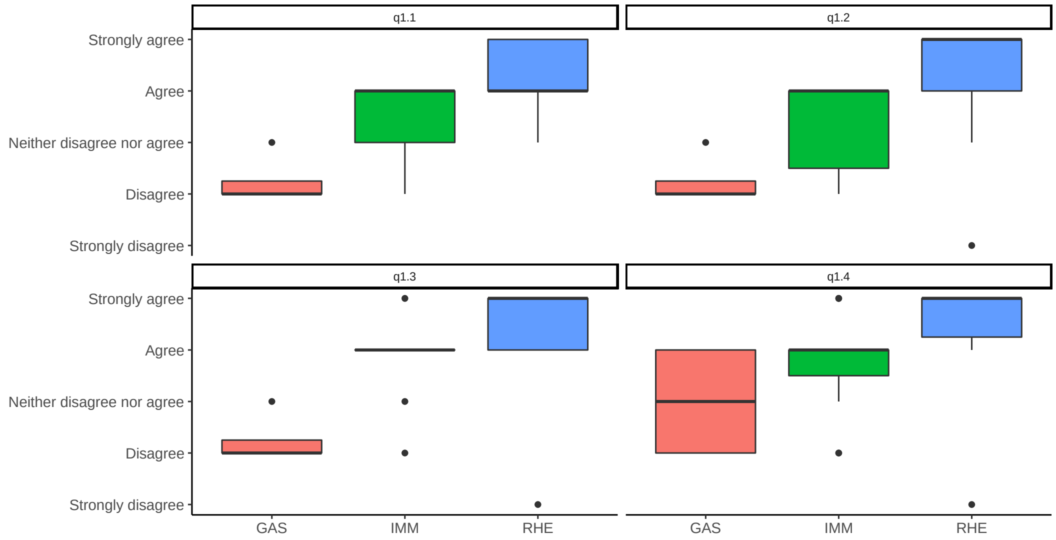

Supplementary Figure S6. Participants’ answers to questions q1.1 and q1.2, by specialty (*n* = 37). *q1.1 = “In general, my level of knowledge about biosimilars is good”, q1.2 = “In general, I feel well informed about biosimilars”, q1.3 = “I feel comfortable talking to my patients about the benefits of biosimilars”, and q1.4 = “I feel comfortable talking to my colleagues about the benefits of biosimilars”.*

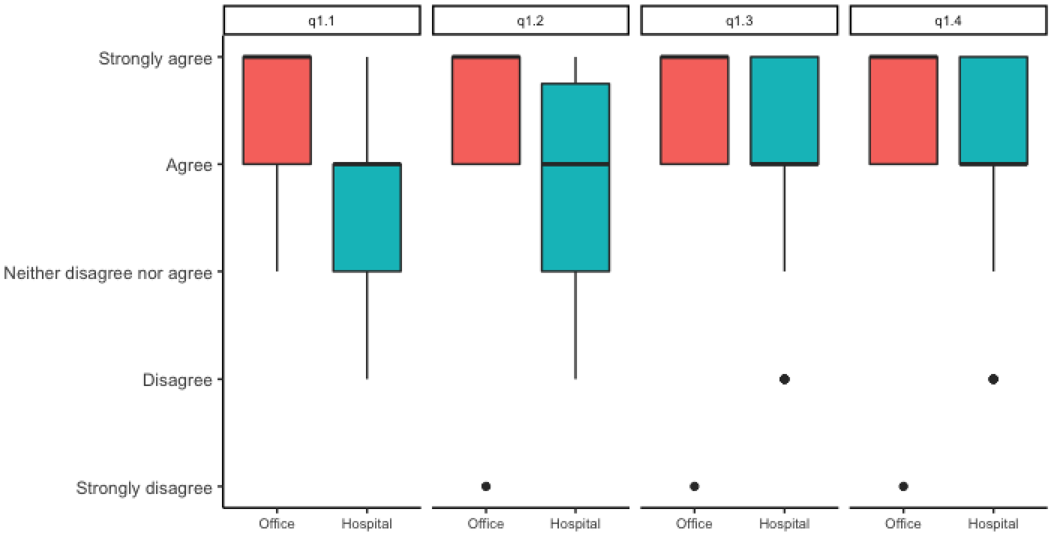

Supplementary Figure S7. Participants' answers to questions q1.1 and q1.2, by place of practice (*n* = 37). *q1.1 = "In general, my level of knowledge about biosimilars is good", q1.2 = "In general, I feel well informed about biosimilars", q1.3 = "I feel comfortable talking to my patients about the benefits of biosimilars", and q1.4 = "I feel comfortable talking to my colleagues about the benefits of biosimilars".*

II.3.2. Question 2

Q2: In the past 12 months, how often have you used the following sources of information about biosimilars?

q2.1 - Self-learning and scientific publications
q2.2 - The pharmaceutical industry (including medical representatives)
q2.3 - The website of the Swiss Agency for Therapeutic Products (swissmedic.ch)
q2.4 - The website of the Federal Office of Public Health (FOPH), including the list of specialties (LS) (bag.admin.ch)
q2.5 - The website for information on medicines (swissmedicinfo.ch)
q2.6 - The website of the Swiss Compendium of Medicines (compendium.ch)
q2.7 - Professional congresses
q2.8 - The media
q2.9 - My medical colleagues
q2.10 - My pharmacist colleagues

II.3.3. Question 3

Q3: Which statements about biosimilars do you think are correct? (*Check all that apply*)

Supplementary Table S14. Questions used to compute the knowledge score and their answer

	Question	Answer
q3.1 -	A biosimilar has an identical amino acid sequence and glycosylation to its reference biologic.	*FALSE*
q3.2 -	A biosimilar is similar to a patent expired reference biologic.	*TRUE*
q3.3 -	A biosimilar has no differences in therapeutic effectiveness from its reference biologic.	*TRUE*
q3.4 -	A biosimilar has no differences in safety (adverse events and serious adverse events) from its reference biologic.	*TRUE*
q3.5 -	A biosimilar does not require preclinical and clinical studies.	*FALSE*
q3.6 -	A biosimilar has the same immunogenicity as its reference biologic.	*TRUE*
q3.7 -	A biosimilar may have minor structural variations in its amino acid sequence from one production batch to another.	*FALSE*

By specialty

Supplementary Table S15. Mean knowledge score, by specialty. Percentages have been rounded and are for information purposes only. Totals do not necessarily add up to 100%.

Specialty	number	mean (sd)
GAS	4	82% (14%)
IMM	11	75% (18%)
RHE	22	72% (16%)
Total	37	74% (16%)

GAS = gastroenterology, IMM = immunoallergology, RHE = rheumatology, sd = standard deviation

Supplementary Table S16. Analysis of variance of the knowledge score, by specialty

Test	p-value
Bartlett	0.85
Levene	0.80

Analysis of mean knowledge score

→ ANOVA:

- F statistic = 0.663,
- p-value = 0.522

Pairwise analysis

→ Tukey's post-hoc test of means

Supplementary Table S17. Results from the Tukey post-hoc test.

Groups	estimate	p value
GAS vs IMM	-0.07	0.763
GAS vs RHE	-0.1	0.511
IMM vs RHE	-0.03	0.857

GAS = gastroenterology, IMM = immunoallergology, RHE = rheumatology

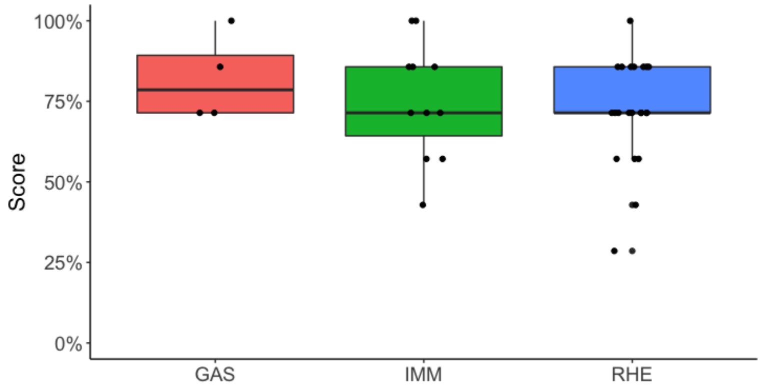

Figure S8. Box plot of participants' knowledge score, by specialty. *GAS = gastroenterology, IMM = immunoallergology, RHE = rheumatology*

Supplementary Table S18. Mean knowledge score, for each question, by specialty

Specialty *n* (%)	q3.1	q3.2	q3.3	q3.4	q3.5	q3.6	q3.7
GAS	2 (50 %)	2 (50 %)	4 (100 %)	4 (100 %)	3 (75 %)	4 (100 %)	4 (100 %)
IMM	8 (73 %)	9 (82 %)	8 (73 %)	8 (73 %)	10 (91 %)	4 (36 %)	11 (100 %)
RHE	19 (51 %)	14 (38 %)	19 (51 %)	19 (51 %)	21 (57 %)	9 (24 %)	10 (27 %)

GAS = gastroenterology, n = number, IMM = immunoallergology, RHE = rheumatology

By place years of cumulative experience

Supplementary Table S19 Mean knowledge score, by years of cumulative experience. Percentages have been rounded and are for information purposes only. Totals do not necessarily add up to 100%.

Years of cumulative experience	number	mean (standard deviation)
< 10 years	18	76% (13%)
Between 10 and 20 years	8	84% (14%)
> 20 years	11	64% (18%)
Total	37	74% (16%)

Supplementary Table S20. Analysis of variance of knowledge score, by years of cumulative experience

Test	p-value
Bartlett	0.38
Levene	0.44

Analysis of mean knowledge score

→ ANOVA:

- F statistic = 4.554,
- p-value = 0.0177

Pairwise analysis

→ Tukey's post-hoc test of means

Supplementary Table S21. Results from the Tukey post-hoc test.

Groups	estimate	p value
< 10 years vs Between 10 years and 20 years	0.08	0.45
< 10 years vs > 20 years	-0.13	0.09
Between 10 years and 20 years vs > 20 years	-0.20	0.02

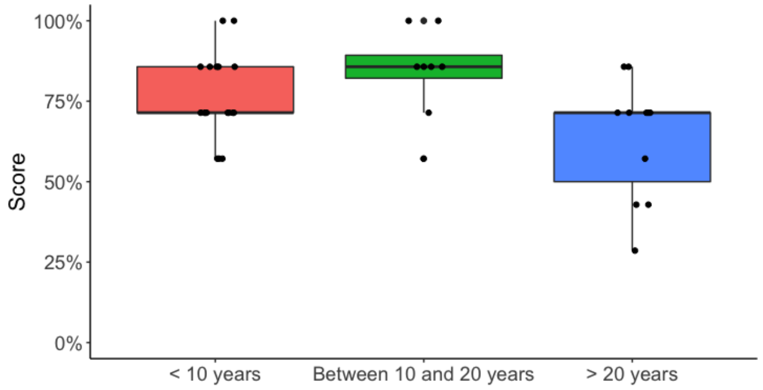

Figure S9. Box plot of participants' knowledge score, by years of cumulative experience

Supplementary Table S22. Mean knowledge score, for each question, by specialty. Percentages have been rounded and are for information purposes only. Totals do not necessarily add up to 100%.

Years of cumulative experience *n* (%)	q3.1	q3.2	q3.3	q3.4	q3.5	q3.6	q3.7
< 10 years	14 (78 %)	10 (56 %)	17 (94 %)	17 (94 %)	15 (83 %)	9 (50 %)	14 (78 %)
Between 10 and 20 years	7 (88 %)	7 (88 %)	7 (88 %)	7 (88 %)	8 (100 %)	4 (50 %)	7 (88 %)
> 20 years	8 (73 %)	8 (73 %)	7 (64 %)	7 (64 %)	11 (100 %)	4 (36 %)	4 (36 %)

n = number

By place of practice

Supplementary Table S23. Mean knowledge score, by place of practice

Place of practice	number	mean (standard deviation)
Office	7	67% (21)
Hospital	30	76% (15)

Supplementary Table S24. Analysis of variance of knowledge score, by place of practice

	p-value
Bartlett	0.32
Levene	0.33

Analysis of mean age

→ Two Sample *t*-test :

- t statistic = -1.2208,
- p-value = 0.2303

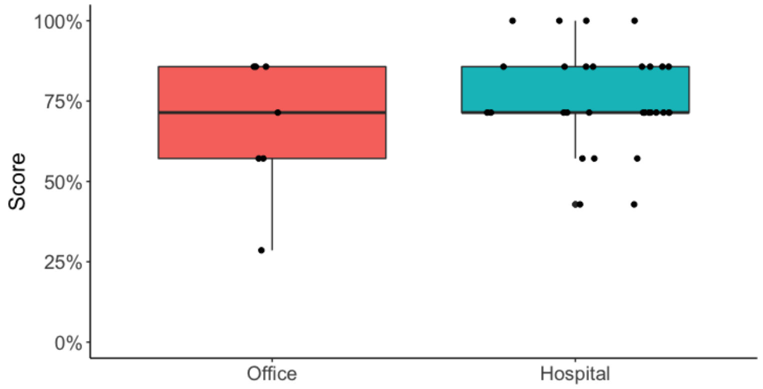

Figure S10. Box plot of participants' knowledge score, by place of practice.

Supplementary Table S25. Mean knowledge score, for each question, by place of practice. Percentages have been rounded and are for information purposes only. Totals do not necessarily add up to 100%.

Place of practice *n* (%)	q3.1	q3.2	q3.3	q3.4	q3.5	q3.6	q3.7
Office	5 (71 %)	5 (71 %)	5 (71 %)	5 (71 %)	7 (100 %)	4 (57 %)	2 (29 %)
Hospital	24 (80 %)	20 (67 %)	26 (87 %)	26 (87 %)	27 (90 %)	13 (43 %)	23 (77 %)

Association between the knowledge score and the ordinal answers to the questions

- KS and "*I explicitly use the term 'biosimilar' when talking to my patients who are going to start a biosimilar or to whom I want to suggest a biosimilar substitution*"

→ Likelihood ratio tests of cumulative link models:

Supplementary Table S26a. Results from the ordinal regression model with question q6.9

	formula:	link:	threshold
modelnull	q6.9 ~ 1	logit	flexible
model1	q6.9 ~ 1 + KS	logit	flexible
	**AIC**	**logLik**	***p* value**
modelnull	112.14	-52.07	
model1	108.30	-49.15	0.02
	**Estimate**	**Standard Error**	
KS	-4.824	2.068	
**Confidence interval**	**2.5%**	**97.5%**	
KS	-9.13	-0.90	

AIC =Akaike information criterion
KS = knowledge score
logit = distribution family of the data
logLik = log-likelihood at the estimated optimum.
model1 = ordinal regression model with knowledge score as the independent variable
modelnull = ordinal regression model without any independent variable

- KS and "*The lack of incentive systems is a barrier to the prescription of biosimilars in Switzerland*"

→ Likelihood ratio tests of cumulative link models:

Supplementary Table S26b. Results from the ordinal regression model with question q7.4

	formula:	link:	threshold
modelnull	q7.4 ~ 1	logit	flexible
model1	q7.4 ~ 1 + KS	logit	flexible
	**AIC**	**logLik**	***p* value**
modelnull	100.34	-46.17	
model1	97.12	-43.56	0.02
	**Estimate**	**Standard Error**	
KS	4.587	4.587	
**Confidence interval**	**2.5%**	**97.5%**	
KS	0.65	0.65	

AIC =Akaike information criterion
KS = knowledge score
logit = distribution family of the data
logLik = log-likelihood at the estimated optimum.
model1 = ordinal regression model with knowledge score as the independent variable
modelnull = ordinal regression model without any independent variable

II.3.4. Question 4

Q4 For which of the following reference biologics (in bold) have you ever prescribed a biosimilar (in italics)?
q4.1 - None of the above
q4.2 - Amgevita® or Hulio® or Hyrimoz® or Imraldi® or Idacio® → (reference biologic: Humira®)
q4.3 - Benepali® or Erelzi® → (reference biologic: Enbrel®)
q4.4 - Inflectra® or Remsima® → (reference biologic: Remicade®)

Supplementary Table S27. Number of biosimilars prescribed , by specialty. Percentages have been rounded and are for information purposes only. Totals do not necessarily add up to 100%.

Specialty *n*	q4.1	q4.2	q4.3	q4.4
GAS	0	1 (3 %)	0	4 (13 %)
IMM	3 (8 %)	0	1 (3 %)	8 (26 %)
RHE	1 (3 %)	14 (45 %)	15 (48 %)	10 (32 %)
Total	4 (11 %)	15 (48 %)	16 (52 %)	22 (71 %)

GAS = gastroenterology, n = number, IMM = immunoallergology, RHE = rheumatology

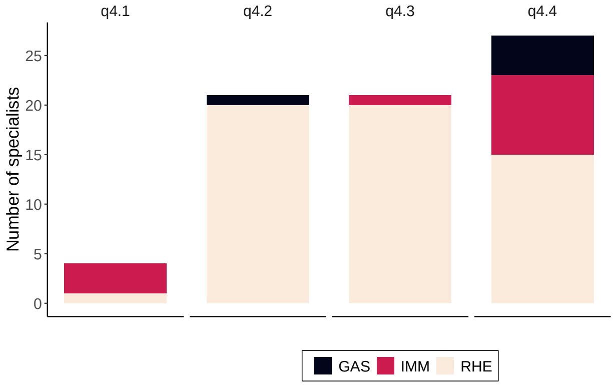

Supplementary Figure S11. Number of biosimilars prescribed , by specialty

II.3.5. Question 5

Q5 In the past 12 months, how often did you prescribe or refill a prescription for any of the biosimilars below?
q5.1 - Amgevita® or Hulio® or Hyrimoz® or Imraldi® or Idacio®
q5.2 - Benepali® or Erelzi®
q5.3 - Inflectra® or Remsima

Supplementary Table S28. Frequency of prescribing biosimilars. Percentages have been rounded and are for information purposes only. Totals do not necessarily add up to 100%.

	q5.1	q5.2	q5.3
Never	14 (42 %)	15 (45 %)	9 (27 %)
Once every 12 months	0	0	6 (18 %)
1-2 times every 6 months	3 (9 %)	6 (18 %)	4 (12 %)
1-2 times every 3 months	6 (18 %)	8 (24 %)	8 (24 %)
1-2 times every month	4 (12 %)	3 (9 %)	5 (15 %)
At least once a week	6 (18 %)	1 (3 %)	1 (3 %)

II.4.Confidence in TNF-α antagonist biosimilars

II.4.1. Question 6

How much do you agree with the following statements? (*strongly disagree, disagree, Neither agree nor disagree, agree, strongly agree*)
q6.1 - “I prescribe a biosimilar to a patient based primarily on my clinical experience”
q6.2 - “Using the literature, I gladly prescribe a biosimilar to patients who have not yet started treatment with the reference biologic”
q6.3 - “Using the literature, I gladly discuss with my patients in remission and under treatment with a reference biologic to propose a substitution with the biosimilar”
q6.4 - “I present information about biosimilars in a positive way in discussions with my patients.”
q6.5 - “When a biosimilar comes on the market, I prefer to wait for the results of substitution in my colleagues' patients before proposing the substitution to my patients”
q6.6 - “I prefer to initiate treatment with the biosimilar in patients who have not yet started treatment with the reference biologic, rather than substituting my patients who are already in remission with the reference biologic”
q6.7 - “I refuse to substitute a biosimilar for the reference biologic in a patient who has had difficulty achieving remission”
q6.8 - “I do not prescribe biosimilars because they are not available in my institution”
q6.9 - “I explicitly use the term 'biosimilar' when talking to my patients who are going to start a biosimilar or to whom I want to suggest a biosimilar substitution”
q6.10 - “My patients who are in remission are willing to substitute their reference biologic with the biosimilar”
q6.11 - “Patients who have not yet started treatment with the reference biologic are willing to start treatment with the biosimilar”
q6.12 - “I have given up prescribing biosimilars because health insurance companies regularly hinder their reimbursement for off-label use”

Refer to Supplementary Table and 26 for data on the following statements “*I feel comfortable talking to my patients about the benefits of biosimilars*” and “*I feel comfortable talking to my colleagues about the benefits of biosimilars*”.

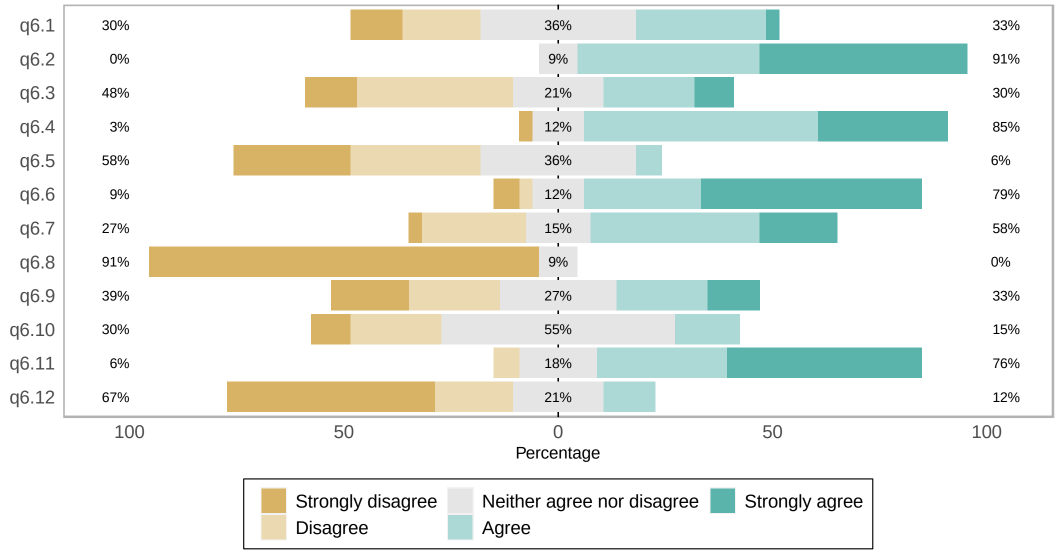

Figure S12. Participants' answer for each item of question "*6. How much do you agree with the following statements?*" (*n* = 33).

II.4.2. <u>Question 7</u>

How much do you agree with the following statements? (*strongly disagree, disagree, Neither agree nor disagree, agree, strongly agree*)

q7.1 - "The prescription of biosimilars is promoted by the Federal Office of Public Health (FOPH)"
q7.2 - "The prescription of biosimilars is promoted by my institution/office"
q7.3 - "Biosimilars are an opportunity to reduce health care costs"
q7.4 - "The lack of incentive systems is a barrier to the prescription of biosimilars in Switzerland"
q7.5 - "I support the routine prescribing of biosimilars in place of the original biologics in patients who have not yet started treatment with the reference biologic."
q7.6 - "I have enough information about the safety of use (adverse events and serious adverse events) of biosimilars to be comfortable with their prescription"
q7.7 - "I have enough information about the therapeutic efficacy of biosimilars to be comfortable with their prescription"
q7.8 - "Patients who have not yet started treatment with the reference biologic should start treatment with a biosimilar, if one exists"
q7.9 - "I have enough time to offer a biosimilar to my patients who have not yet started treatment with the reference biologic and to explain the rationale for it"
q7.10 - "I have enough time to propose a biosimilar substitution to my patients who are already on a reference biologic and in remission and to explain the rationale for it"
q7.11 - "Prescribing a biosimilar gives me confidence in the therapeutic management of my patients who are already on a reference biologic and in remission"
q7.12 - "Prescribing a biosimilar gives me confidence in the therapeutic management of my patients who have not yet started their treatment with the reference biologic"
q7.13 - "The success of the substitution of a biologic by its biosimilar depends mainly on the physician-patient relationship"

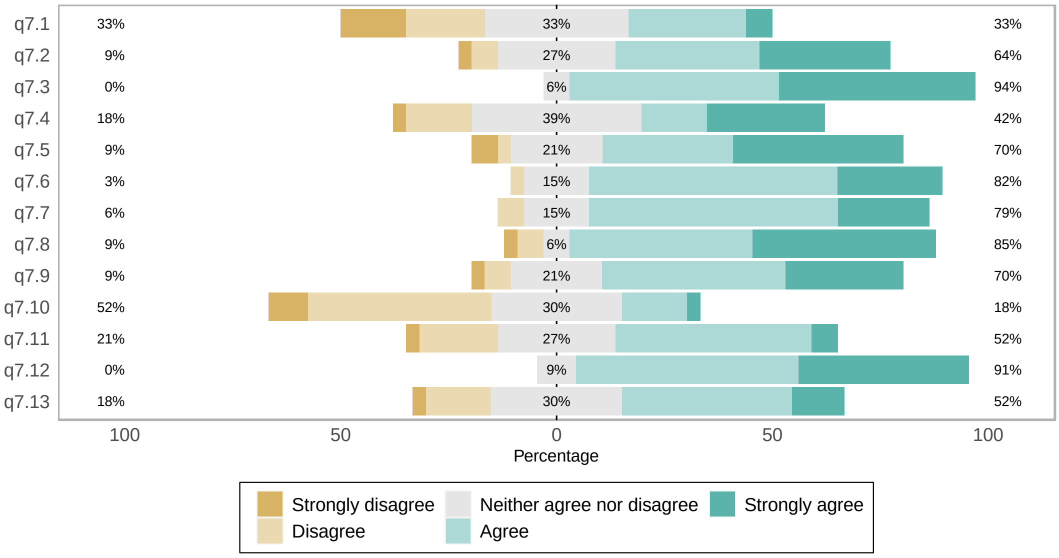

Figure S13. *Participants' answer for each item of question "7.How much do you agree with the following statements?" (n = 33).*

Supplementary Table S29. Ordinal data for questions q1.1-4, by specialty, years of cumulative experience and place of practice. Percentages have been rounded and are for information purposes only. Totals do not necessarily add up to 100%.

**q1.1**	**GAS**	**IMM**	**RHE**	**< 10 years**	**Between 10 and 20 years**	**> 20 years**	**Office**	**Hospital**
Strongly disagree	0	0	0	0	0	0	0	0
Disagree	3 (8 %)	2 (5 %)	0	4 (11 %)	1 (3 %)	0	0	5 (14 %)
Neither disagree nor agree	1 (3 %)	3 (8 %)	3 (8 %)	4 (11 %)	1 (3 %)	2 (5 %)	1 (3 %)	6 (16 %)
Agree	0	6 (16 %)	10 (27 %)	8 (22 %)	3 (8 %)	5 (14 %)	2 (5 %)	14 (38 %)
Strongly agree	0	0	9 (24 %)	2 (5 %)	3 (8 %)	4 (11 %)	4 (11 %)	5 (14 %)
**q1.2**	**GAS**	**IMM**	**RHE**	**< 10 years**	**Between 10 and 20 years**	**> 20 years**	**Office**	**Hospital**
Strongly disagree	0	0	1 (3 %)	0	0	1 (3 %)	1 (3 %)	0
Disagree	3 (8 %)	3 (8 %)	0	5 (14 %)	1 (3 %)	0	0	6 (16 %)
Neither disagree nor agree	1 (3 %)	2 (5 %)	1 (3 %)	3 (8 %)	0	1 (3 %)	0	4 (11 %)
Agree	0	6 (16 %)	8 (22 %)	6 (16 %)	4 (11 %)	4 (11 %)	2 (5 %)	12 (32 %)
Strongly agree	0	0	12 (32 %)	4 (11 %)	3 (8 %)	5 (14 %)	4 (11 %)	8 (22 %)
**q1.3**	**GAS**	**IMM**	**RHE**	**< 10 years**	**Between 10 and 20 years**	**> 20 years**	**Office**	**Hospital**
Strongly disagree	0	0	1 (3 %)	0	0	1 (3 %)	1 (3 %)	0
Disagree	3 (8 %)	1 (3 %)	0	3 (8 %)	1 (3 %)	0	0	4 (11 %)
Neither disagree nor agree	1 (3 %)	1 (3 %)	0	2 (5 %)	0	0	0	2 (5 %)
Agree	0	8 (22 %)	8 (22 %)	9 (24 %)	2 (5 %)	5 (14 %)	2 (5 %)	14 (38 %)
Strongly agree	0	1 (3 %)	13 (35 %)	4 (11 %)	5 (14 %)	5 (14 %)	4 (11 %)	10 (27 %)
**q1.4**	**GAS**	**IMM**	**RHE**	**< 10 years**	**Between 10 and 20 years**	**> 20 years**	**Office**	**Hospital**
Strongly disagree	0	0	1 (3 %)	0	0	1 (3 %)	1 (3 %)	0
Disagree	2 (5 %)	2 (5 %)	0	3 (8 %)	1 (3 %)	0	0	4 (11 %)
Neither disagree nor agree	0	1 (3 %)	0	1 (3 %)	0	0	0	1 (3 %)
Agree	2 (5 %)	6 (16 %)	5 (14 %)	7 (19 %)	2 (5 %)	4 (11 %)	2 (5 %)	11 (30 %)
Strongly agree	0	2 (5 %)	16 (43 %)	7 (19 %)	5 (14 %)	6 (16 %)	4 (11 %)	14 (38 %)

GAS = gastroenterology, IMM = immunoallergology, RHE = rheumatology

Supplementary Table S30. Ordinal data for questions q2.1-10. Percentages have been rounded and are for information purposes only. Totals do not necessarily add up to 100%.

	**q2.1**	**q2.2**	**q2.3**	**q2.4**	**q2.5**	**q2.6**	**q2.7**	**q2.8**	**q2.9**	**q2.10**
Never	3 (8 %)	9 (24 %)	20 (54 %)	17 (46 %)	22 (59 %)	3 (8 %)	5 (14 %)	30 (81 %)	7 (19 %)	22 (59 %)
Once every 12 months	4 (11 %)	7 (19 %)	5 (14 %)	7 (19 %)	5 (14 %)	5 (14 %)	10 (27 %)	5 (14 %)	6 (16 %)	6 (16 %)
1-2 times every 6 months	10 (27 %)	10 (27 %)	5 (14 %)	3 (8 %)	2 (5 %)	8 (22 %)	17 (46 %)	2 (5 %)	11 (30 %)	8 (22 %)
1-2 times every 3 months	11 (30 %)	8 (22 %)	2 (5 %)	3 (8 %)	2 (5 %)	8 (22 %)	4 (11 %)	0	3 (8 %)	1 (3 %)
1-2 times every month	8 (22 %)	2 (5 %)	3 (8 %)	3 (8 %)	3 (8 %)	5 (14 %)	1 (3 %)	0	6 (16 %)	0
At least once a week	1 (3 %)	1 (3 %)	2 (5 %)	4 (11 %)	3 (8 %)	8 (22 %)	0	0	4 (11 %)	0

Supplementary Table S31. Ordinal data for questions q2.1-10, by specialty, years of cumulative experience and place of practice (1/2).

**q2.1**	**GAS**	**IMM**	**RHE**	**< 10 years**	**Between 10 and 20 years**	**> 20 years**	**Office**	**Hospital**
Never	2 (5 %)	0	1 (3 %)	2 (5 %)	1 (3 %)	0	0	3 (8 %)
Once every 12 months	2 (5 %)	1 (3 %)	1 (3 %)	2 (5 %)	2 (5 %)	0	0	4 (11 %)
1-2 times every 6 months	0	4 (11 %)	6 (16 %)	5 (14 %)	2 (5 %)	3 (8 %)	2 (5 %)	8 (22 %)
1-2 times every 3 months	0	3 (8 %)	8 (22 %)	4 (11 %)	2 (5 %)	5 (14 %)	2 (5 %)	9 (24 %)
1-2 times every month	0	3 (8 %)	5 (14 %)	5 (14 %)	0	3 (8 %)	2 (5 %)	6 (16 %)
At least once a week	0	0	1 (3 %)	0	1 (3 %)	0	1 (3 %)	0

**q2.2**	**GAS**	**IMM**	**RHE**	**< 10 years**	**Between 10 and 20 years**	**> 20 years**	**Office**	**Hospital**
Never	2 (5 %)	5 (14 %)	2 (5 %)	6 (16 %)	2 (5 %)	1 (3 %)	1 (3 %)	8 (22 %)
Once every 12 months	1 (3 %)	3 (8 %)	3 (8 %)	4 (11 %)	0	3 (8 %)	0	7 (19 %)
1-2 times every 6 months	1 (3 %)	1 (3 %)	8 (22 %)	3 (8 %)	3 (8 %)	4 (11 %)	2 (5 %)	8 (22 %)
1-2 times every 3 months	0	2 (5 %)	6 (16 %)	4 (11 %)	2 (5 %)	2 (5 %)	2 (5 %)	6 (16 %)
1-2 times every month	0	0	2 (5 %)	1 (3 %)	0	1 (3 %)	1 (3 %)	1 (3 %)
At least once a week	0	0	1 (3 %)	0	1 (3 %)	0	1 (3 %)	0

**q2.3**	**GAS**	**IMM**	**RHE**	**< 10 years**	**Between 10 and 20 years**	**> 20 years**	**Office**	**Hospital**
Never	2 (5 %)	4 (11 %)	14 (38 %)	7 (19 %)	7 (19 %)	6 (16 %)	3 (8 %)	17 (46 %)
Once every 12 months	1 (3 %)	1 (3 %)	3 (8 %)	3 (8 %)	0	2 (5 %)	1 (3 %)	4 (11 %)
1-2 times every 6 months	0	4 (11 %)	1 (3 %)	3 (8 %)	1 (3 %)	1 (3 %)	0	5 (14 %)
1-2 times every 3 months	0	1 (3 %)	1 (3 %)	1 (3 %)	0	1 (3 %)	1 (3 %)	1 (3 %)
1-2 times every month	0	1 (3 %)	2 (5 %)	3 (8 %)	0	0	1 (3 %)	2 (5 %)
At least once a week	1 (3 %)	0	1 (3 %)	1 (3 %)	0	1 (3 %)	1 (3 %)	1 (3 %)

**q2.4**	**GAS**	**IMM**	**RHE**	**< 10 years**	**Between 10 and 20 years**	**> 20 years**	**Office**	**Hospital**
Never	3 (8 %)	4 (11 %)	10 (27 %)	7 (19 %)	4 (11 %)	6 (16 %)	4 (11 %)	13 (35 %)
Once every 12 months	1 (3 %)	3 (8 %)	3 (8 %)	3 (8 %)	0	4 (11 %)	0	7 (19 %)
1-2 times every 6 months	0	1 (3 %)	2 (5 %)	2 (5 %)	1 (3 %)	0	0	3 (8 %)
1-2 times every 3 months	0	2 (5 %)	1 (3 %)	2 (5 %)	1 (3 %)	0	0	3 (8 %)
1-2 times every month	0	1 (3 %)	2 (5 %)	3 (8 %)	0	0	1 (3 %)	2 (5 %)
At least once a week	0	0	4 (11 %)	1 (3 %)	2 (5 %)	1 (3 %)	2 (5 %)	2 (5 %)

**q2.5**	**GAS**	**IMM**	**RHE**	**< 10 years**	**Between 10 and 20 years**	**> 20 years**	**Office**	**Hospital**
Never	2 (5 %)	5 (14 %)	15 (41 %)	8 (22 %)	6 (16 %)	8 (22 %)	6 (16 %)	16 (43 %)
Once every 12 months	1 (3 %)	3 (8 %)	1 (3 %)	3 (8 %)	0	2 (5 %)	0	5 (14 %)
1-2 times every 6 months	0	1 (3 %)	1 (3 %)	1 (3 %)	1 (3 %)	0	0	2 (5 %)
1-2 times every 3 months	0	1 (3 %)	1 (3 %)	1 (3 %)	0	1 (3 %)	0	2 (5 %)
1-2 times every month	1 (3 %)	1 (3 %)	1 (3 %)	3 (8 %)	0	0	1 (3 %)	2 (5 %)
At least once a week	0	0	3 (8 %)	2 (5 %)	1 (3 %)	0	0	3 (8 %)

GAS = gastroenterology, IMM = immunoallergology, RHE = rheumatology

Supplementary Table S32. Ordinal data for questions q2.1-10, by specialty, years of cumulative experience and place of practice (2/2). Percentages have been rounded and are for information purposes only. Totals do not necessarily add up to 100%.

**q2.6**	**GAS**	**IMM**	**RHE**	**< 10 years**	**Between 10 and 20 years**	**> 20 years**	**Office**	**Hospital**
Never	0	0	3 (8 %)	1 (3 %)	2 (5 %)	0	0	3 (8 %)
Once every 12 months	2 (5 %)	2 (5 %)	1 (3 %)	5 (14 %)	0	0	0	5 (14 %)
1-2 times every 6 months	0	2 (5 %)	6 (16 %)	1 (3 %)	2 (5 %)	5 (14 %)	2 (5 %)	6 (16 %)
1-2 times every 3 months	1 (3 %)	4 (11 %)	3 (8 %)	3 (8 %)	3 (8 %)	2 (5 %)	0	8 (22 %)
1-2 times every month	0	0	5 (14 %)	1 (3 %)	0	4 (11 %)	3 (8 %)	2 (5 %)
At least once a week	1 (3 %)	3 (8 %)	4 (11 %)	7 (19 %)	1 (3 %)	0	2 (5 %)	6 (16 %)
**q2.7**	**GAS**	**IMM**	**RHE**	**< 10 years**	**Between 10 and 20 years**	**> 20 years**	**Office**	**Hospital**
Never	2 (5 %)	1 (3 %)	2 (5 %)	2 (5 %)	2 (5 %)	1 (3 %)	1 (3 %)	4 (11 %)
Once every 12 months	1 (3 %)	2 (5 %)	7 (19 %)	4 (11 %)	4 (11 %)	2 (5 %)	0	10 (27 %)
1-2 times every 6 months	1 (3 %)	7 (19 %)	9 (24 %)	9 (24 %)	1 (3 %)	7 (19 %)	4 (11 %)	13 (35 %)
1-2 times every 3 months	0	1 (3 %)	3 (8 %)	2 (5 %)	1 (3 %)	1 (3 %)	2 (5 %)	2 (5 %)
1-2 times every month	0	0	1 (3 %)	1 (3 %)	0	0	0	1 (3 %)
At least once a week	0	0	0	0	0	0	0	0
**q2.8**	**GAS**	**IMM**	**RHE**	**< 10 years**	**Between 10 and 20 years**	**> 20 years**	**Office**	**Hospital**
Never	3 (8 %)	10 (27 %)	17 (46 %)	15 (41 %)	7 (19 %)	8 (22 %)	5 (14 %)	25 (68 %)
Once every 12 months	1 (3 %)	1 (3 %)	3 (8 %)	2 (5 %)	1 (3 %)	2 (5 %)	1 (3 %)	4 (11 %)
1-2 times every 6 months	0	0	2 (5 %)	1 (3 %)	0	1 (3 %)	1 (3 %)	1 (3 %)
1-2 times every 3 months	0	0	0	0	0	0	0	0
1-2 times every month	0	0	0	0	0	0	0	0
At least once a week	0	0	0	0	0	0	0	0
**q2.9**	**GAS**	**IMM**	**RHE**	**< 10 years**	**Between 10 and 20 years**	**> 20 years**	**Office**	**Hospital**
Never	0	0	7 (19 %)	2 (5 %)	2 (5 %)	3 (8 %)	3 (8 %)	4 (11 %)
Once every 12 months	1 (3 %)	1 (3 %)	4 (11 %)	2 (5 %)	1 (3 %)	3 (8 %)	0	6 (16 %)
1-2 times every 6 months	2 (5 %)	4 (11 %)	5 (14 %)	4 (11 %)	4 (11 %)	3 (8 %)	3 (8 %)	8 (22 %)
1-2 times every 3 months	1 (3 %)	2 (5 %)	0	3 (8 %)	0	0	0	3 (8 %)
1-2 times every month	0	2 (5 %)	4 (11 %)	4 (11 %)	0	2 (5 %)	1 (3 %)	5 (14 %)
At least once a week	0	2 (5 %)	2 (5 %)	3 (8 %)	1 (3 %)	0	0	4 (11 %)
**q2.10**	**GAS**	**IMM**	**RHE**	**< 10 years**	**Between 10 and 20 years**	**> 20 years**	**Office**	**Hospital**
Never	3 (8 %)	3 (8 %)	16 (43 %)	9 (24 %)	6 (16 %)	7 (19 %)	6 (16 %)	16 (43 %)
Once every 12 months	0	4 (11 %)	2 (5 %)	3 (8 %)	2 (5 %)	1 (3 %)	0	6 (16 %)
1-2 times every 6 months	1 (3 %)	4 (11 %)	3 (8 %)	5 (14 %)	0	3 (8 %)	1 (3 %)	7 (19 %)
1-2 times every 3 months	0	0	1 (3 %)	1 (3 %)	0	0	0	1 (3 %)
1-2 times every month	0	0	0	0	0	0	0	0
At least once a week	0	0	0	0	0	0	0	0

GAS = gastroenterology, IMM = immunoallergology, RHE = rheumatology

Supplementary Table S33. Ordinal data for questions q6.1-12. Percentages have been rounded and are for information purposes only. Totals do not necessarily add up to 100%.

	q6.1	q6.2	q6.3	q6.4	q6.5	q6.6	q6.7	q6.8	q6.9	q6.10	q6.11	q6.12
Strongly disagree	4 (12 %)	0	4 (12 %)	1 (3 %)	9 (27 %)	2 (6 %)	1 (3 %)	30 (91 %)	6 (18 %)	3 (9 %)	0	16 (48 %)
Disagree	6 (18 %)	0	12 (36 %)	0	10 (30 %)	1 (3 %)	8 (24 %)	0	7 (21 %)	7 (21 %)	2 (6 %)	6 (18 %)
Neither disagree nor agree	12 (36 %)	3 (9 %)	7 (21 %)	4 (12 %)	12 (36 %)	4 (12 %)	5 (15 %)	3 (9 %)	9 (27 %)	18 (55 %)	6 (18 %)	7 (21 %)
Agree	10 (30 %)	14 (42 %)	7 (21 %)	18 (55 %)	2 (6 %)	9 (27 %)	13 (39 %)	0	7 (21 %)	5 (15 %)	10 (30 %)	4 (12 %)
Strongly agree	1 (3 %)	16 (48 %)	3 (9 %)	10 (30 %)	0	17 (52 %)	6 (18 %)	0	4 (12 %)	0	15 (45 %)	0

Supplementary Table S34. Ordinal data for questions q6.1-12, by specialty, years of cumulative experience and place of practice (1/3).

**q6.1**	**GAS**	**IMM**	**RHE**	**< 10 years**	**Between 10 and 20 years**	**> 20 years**	**Office**	**Hospital**
Strongly disagree	1 (3 %)	1 (3 %)	2 (6 %)	2 (6 %)	1 (3 %)	1 (3 %)	0	4 (12 %)
Disagree	1 (3 %)	2 (6 %)	3 (9 %)	3 (9 %)	1 (3 %)	2 (6 %)	1 (3 %)	5 (15 %)
Neither disagree nor agree	2 (6 %)	3 (9 %)	7 (21 %)	7 (21 %)	2 (6 %)	3 (9 %)	2 (6 %)	10 (30 %)
Agree	0	2 (6 %)	8 (24 %)	4 (12 %)	2 (6 %)	4 (12 %)	4 (12 %)	6 (18 %)
Strongly agree	0	0	1 (3 %)	0	1 (3 %)	0	0	1 (3 %)
**q6.2**	**GAS**	**IMM**	**RHE**	**< 10 years**	**Between 10 and 20 years**	**> 20 years**	**Office**	**Hospital**
Strongly disagree	0	0	0	0	0	0	0	0
Disagree	0	0	0	0	0	0	0	0
Neither disagree nor agree	2 (6 %)	1 (3 %)	0	2 (6 %)	1 (3 %)	0	0	3 (9 %)
Agree	2 (6 %)	6 (18 %)	6 (18 %)	7 (21 %)	1 (3 %)	6 (18 %)	4 (12 %)	10 (30 %)
Strongly agree	0	1 (3 %)	15 (45 %)	7 (21 %)	5 (15 %)	4 (12 %)	3 (9 %)	13 (39 %)
**q6.3**	**GAS**	**IMM**	**RHE**	**< 10 years**	**Between 10 and 20 years**	**> 20 years**	**Office**	**Hospital**
Strongly disagree	2 (6 %)	0	2 (6 %)	1 (3 %)	1 (3 %)	2 (6 %)	2 (6 %)	2 (6 %)
Disagree	0	5 (15 %)	7 (21 %)	6 (18 %)	3 (9 %)	3 (9 %)	1 (3 %)	11 (33 %)
Neither disagree nor agree	2 (6 %)	3 (9 %)	2 (6 %)	5 (15 %)	1 (3 %)	1 (3 %)	0	7 (21 %)
Agree	0	0	7 (21 %)	2 (6 %)	1 (3 %)	4 (12 %)	3 (9 %)	4 (12 %)
Strongly agree	0	0	3 (9 %)	2 (6 %)	1 (3 %)	0	1 (3 %)	2 (6 %)
**q6.4**	**GAS**	**IMM**	**RHE**	**< 10 years**	**Between 10 and 20 years**	**> 20 years**	**Office**	**Hospital**
Strongly disagree	0	1 (3 %)	0	1 (3 %)	0	0	0	1 (3 %)
Disagree	0	0	0	0	0	0	0	0
Neither disagree nor agree	2 (6 %)	2 (6 %)	0	3 (9 %)	1 (3 %)	0	0	4 (12 %)
Agree	2 (6 %)	3 (9 %)	13 (39 %)	5 (15 %)	3 (9 %)	10 (30 %)	5 (15 %)	13 (39 %)
Strongly agree	0	2 (6 %)	8 (24 %)	7 (21 %)	3 (9 %)	0	2 (6 %)	8 (24 %)

GAS = gastroenterology, IMM = immunoallergology, RHE = rheumatology

Supplementary Table S35. Ordinal data for questions q6.1-12, by specialty, years of cumulative experience and place of practice (2/3).

**q6.5**	**GAS**	**IMM**	**RHE**	**< 10 years**	**Between 10 and 20 years**	**> 20 years**	**Office**	**Hospital**
Strongly disagree	1 (3 %)	2 (6 %)	6 (18 %)	4 (12 %)	2 (6 %)	3 (9 %)	3 (9 %)	6 (18 %)
Disagree	1 (3 %)	2 (6 %)	7 (21 %)	4 (12 %)	3 (9 %)	3 (9 %)	1 (3 %)	9 (27 %)
Neither disagree nor agree	1 (3 %)	3 (9 %)	8 (24 %)	6 (18 %)	2 (6 %)	4 (12 %)	3 (9 %)	9 (27 %)
Agree	1 (3 %)	1 (3 %)	0	2 (6 %)	0	0	0	2 (6 %)
Strongly agree	0	0	0	0	0	0	0	0
**q6.6**	**GAS**	**IMM**	**RHE**	**< 10 years**	**Between 10 and 20 years**	**> 20 years**	**Office**	**Hospital**
Strongly disagree	0	0	2 (6 %)	0	0	2 (6 %)	2 (6 %)	0
Disagree	1 (3 %)	0	0	1 (3 %)	0	0	0	1 (3 %)
Neither disagree nor agree	0	2 (6 %)	2 (6 %)	3 (9 %)	0	1 (3 %)	0	4 (12 %)
Agree	2 (6 %)	3 (9 %)	4 (12 %)	3 (9 %)	4 (12 %)	2 (6 %)	2 (6 %)	7 (21 %)
Strongly agree	1 (3 %)	3 (9 %)	13 (39 %)	9 (27 %)	3 (9 %)	5 (15 %)	3 (9 %)	14 (42 %)
**q6.7**	**GAS**	**IMM**	**RHE**	**< 10 years**	**Between 10 and 20 years**	**> 20 years**	**Office**	**Hospital**
Strongly disagree	0	0	1 (3 %)	0	0	1 (3 %)	1 (3 %)	0
Disagree	1 (3 %)	2 (6 %)	5 (15 %)	4 (12 %)	1 (3 %)	3 (9 %)	3 (9 %)	5 (15 %)
Neither disagree nor agree	1 (3 %)	2 (6 %)	2 (6 %)	4 (12 %)	1 (3 %)	0	0	5 (15 %)
Agree	1 (3 %)	4 (12 %)	8 (24 %)	6 (18 %)	4 (12 %)	3 (9 %)	2 (6 %)	11 (33 %)
Strongly agree	1 (3 %)	0	5 (15 %)	2 (6 %)	1 (3 %)	3 (9 %)	1 (3 %)	5 (15 %)
**q6.8**	**GAS**	**IMM**	**RHE**	**< 10 years**	**Between 10 and 20 years**	**> 20 years**	**Office**	**Hospital**
Strongly disagree	3 (9 %)	6 (18 %)	21 (64 %)	13 (39 %)	7 (21 %)	10 (30 %)	7 (21 %)	23 (70 %)
Disagree	0	0	0	0	0	0	0	0
Neither disagree nor agree	1 (3 %)	2 (6 %)	0	3 (9 %)	0	0	0	3 (9 %)
Agree	0	0	0	0	0	0	0	0
Strongly agree	0	0	0	0	0	0	0	0
**q6.9**	**GAS**	**IMM**	**RHE**	**< 10 years**	**Between 10 and 20 years**	**> 20 years**	**Office**	**Hospital**
Strongly disagree	2 (6 %)	1 (3 %)	3 (9 %)	3 (9 %)	1 (3 %)	2 (6 %)	0	6 (18 %)
Disagree	0	3 (9 %)	4 (12 %)	4 (12 %)	2 (6 %)	1 (3 %)	1 (3 %)	6 (18 %)
Neither disagree nor agree	2 (6 %)	2 (6 %)	5 (15 %)	5 (15 %)	2 (6 %)	2 (6 %)	2 (6 %)	7 (21 %)
Agree	0	2 (6 %)	5 (15 %)	2 (6 %)	1 (3 %)	4 (12 %)	3 (9 %)	4 (12 %)
Strongly agree	0	0	4 (12 %)	2 (6 %)	1 (3 %)	1 (3 %)	1 (3 %)	3 (9 %)
**q6.10**	**GAS**	**IMM**	**RHE**	**< 10 years**	**Between 10 and 20 years**	**> 20 years**	**Office**	**Hospital**
Strongly disagree	0	1 (3 %)	2 (6 %)	0	2 (6 %)	1 (3 %)	1 (3 %)	2 (6 %)
Disagree	1 (3 %)	1 (3 %)	5 (15 %)	4 (12 %)	1 (3 %)	2 (6 %)	2 (6 %)	5 (15 %)
Neither disagree nor agree	3 (9 %)	4 (12 %)	11 (33 %)	9 (27 %)	3 (9 %)	6 (18 %)	2 (6 %)	16 (48 %)
Agree	0	2 (6 %)	3 (9 %)	3 (9 %)	1 (3 %)	1 (3 %)	2 (6 %)	3 (9 %)
Strongly agree	0	0	0	0	0	0	0	0

GAS = gastroenterology, IMM = immunoallergology, RHE = rheumatology

Supplementary Table S36. Ordinal data for questions q6.1-12, by specialty, years of cumulative experience and place of practice (3/3). Percentages have been rounded and are for information purposes only. Totals do not necessarily add up to 100%.

**q6.11**	**GAS**	**IMM**	**RHE**	**< 10 years**	**Between 10 and 20 years**	**> 20 years**	**Office**	**Hospital**
Strongly disagree	0	0	0	0	0	0	0	0
Disagree	0	1 (3 %)	1 (3 %)	1 (3 %)	0	1 (3 %)	0	2 (6 %)
Neither disagree nor agree	3 (9 %)	1 (3 %)	2 (6 %)	3 (9 %)	1 (3 %)	2 (6 %)	2 (6 %)	4 (12 %)
Agree	1 (3 %)	3 (9 %)	6 (18 %)	5 (15 %)	2 (6 %)	3 (9 %)	2 (6 %)	8 (24 %)
Strongly agree	0	3 (9 %)	12 (36 %)	7 (21 %)	4 (12 %)	4 (12 %)	3 (9 %)	12 (36 %)
**q6.12**	**GAS**	**IMM**	**RHE**	**< 10 years**	**Between 10 and 20 years**	**> 20 years**	**Office**	**Hospital**
Strongly disagree	2 (6 %)	2 (6 %)	12 (36 %)	6 (18 %)	4 (12 %)	6 (18 %)	7 (21 %)	9 (27 %)
Disagree	0	4 (12 %)	2 (6 %)	4 (12 %)	1 (3 %)	1 (3 %)	0	6 (18 %)
Neither disagree nor agree	2 (6 %)	1 (3 %)	4 (12 %)	5 (15 %)	0	2 (6 %)	0	7 (21 %)
Agree	0	1 (3 %)	3 (9 %)	1 (3 %)	2 (6 %)	1 (3 %)	0	4 (12 %)
Strongly agree	0	0	0	0	0	0	0	0

GAS = gastroenterology, IMM = immunoallergology, RHE = rheumatology

Supplementary Table S37. Ordinal data for questions q7.1-13. Percentages have been rounded and are for information purposes only. Totals do not necessarily add up to 100%.

	**q7.1**	**q7.2**	**q7.3**	**q7.4**	**q7.5**	**q7.6**	**q7.7**	**q7.8**	**q7.9**	**q7.10**	**q7.11**	**q7.12**	**q7.13**
Strongly disagree	5 (15 %)	1 (3 %)	0	1 (3 %)	2 (6 %)	0	0	1 (3 %)	1 (3 %)	3 (9 %)	1 (3 %)	0	1 (3 %)
Disagree	6 (18 %)	2 (6 %)	0	5 (15 %)	1 (3 %)	1 (3 %)	2 (6 %)	2 (6 %)	2 (6 %)	14 (42 %)	6 (18 %)	0	5 (15 %)
Neither disagree nor agree	11 (33 %)	9 (27 %)	2 (6 %)	13 (39 %)	7 (21 %)	5 (15 %)	5 (15 %)	2 (6 %)	7 (21 %)	10 (30 %)	9 (27 %)	3 (9 %)	10 (30 %)
Agree	9 (27 %)	11 (33 %)	16 (48 %)	5 (15 %)	10 (30 %)	19 (58 %)	19 (58 %)	14 (42 %)	14 (42 %)	5 (15 %)	15 (45 %)	17 (52 %)	13 (39 %)
Strongly agree	2 (6 %)	10 (30 %)	15 (45 %)	9 (27 %)	13 (39 %)	8 (24 %)	7 (21 %)	14 (42 %)	9 (27 %)	1 (3 %)	2 (6 %)	13 (39 %)	4 (12 %)

Supplementary Table S38. Ordinal data for questions q7.1-13, by specialty, years of cumulative experience and place of practice (1/3)

**q7.1**	**GAS**	**IMM**	**RHE**	**< 10 years**	**Between 10 and 20 years**	**> 20 years**	**Office**	**Hospital**
Strongly disagree	2 (6 %)	0	3 (9 %)	1 (3 %)	2 (6 %)	2 (6 %)	1 (3 %)	4 (12 %)
Disagree	1 (3 %)	2 (6 %)	3 (9 %)	4 (12 %)	1 (3 %)	1 (3 %)	2 (6 %)	4 (12 %)
Neither disagree nor agree	1 (3 %)	4 (12 %)	6 (18 %)	6 (18 %)	2 (6 %)	3 (9 %)	1 (3 %)	10 (30 %)
Agree	0	2 (6 %)	7 (21 %)	4 (12 %)	2 (6 %)	3 (9 %)	2 (6 %)	7 (21 %)
Strongly agree	0	0	2 (6 %)	1 (3 %)	0	1 (3 %)	1 (3 %)	1 (3 %)
**q7.2**	**GAS**	**IMM**	**RHE**	**< 10 years**	**Between 10 and 20 years**	**> 20 years**	**Office**	**Hospital**
Strongly disagree	1 (3 %)	0	0	0	1 (3 %)	0	0	1 (3 %)
Disagree	1 (3 %)	0	1 (3 %)	1 (3 %)	0	1 (3 %)	1 (3 %)	1 (3 %)
Neither disagree nor agree	0	4 (12 %)	5 (15 %)	4 (12 %)	2 (6 %)	3 (9 %)	3 (9 %)	6 (18 %)
Agree	1 (3 %)	4 (12 %)	6 (18 %)	6 (18 %)	2 (6 %)	3 (9 %)	1 (3 %)	10 (30 %)
Strongly agree	1 (3 %)	0	9 (27 %)	5 (15 %)	2 (6 %)	3 (9 %)	2 (6 %)	8 (24 %)

GAS = gastroenterology, IMM = immunoallergology, RHE = rheumatology

Supplementary Table S39. Ordinal data for questions q7.1-13, by specialty, years of cumulative experience and place of practice (2/3)

q7.3	GAS	IMM	RHE	< 10 years	Between 10 and 20 years	> 20 years	Office	Hospital
Strongly disagree	0	0	0	0	0	0	0	0
Disagree	0	0	0	0	0	0	0	0
Neither disagree nor agree	0	2 (6 %)	0	2 (6 %)	0	0	0	2 (6 %)
Agree	3 (9 %)	3 (9 %)	10 (30 %)	6 (18 %)	6 (18 %)	4 (12 %)	4 (12 %)	12 (36 %)
Strongly agree	1 (3 %)	3 (9 %)	11 (33 %)	8 (24 %)	1 (3 %)	6 (18 %)	3 (9 %)	12 (36 %)
**q7.4**	**GAS**	**IMM**	**RHE**	**< 10 years**	**Between 10 and 20 years**	**> 20 years**	**Office**	**Hospital**
Strongly disagree	0	0	1 (3 %)	0	0	1 (3 %)	1 (3 %)	0
Disagree	0	2 (6 %)	3 (9 %)	3 (9 %)	1 (3 %)	1 (3 %)	1 (3 %)	4 (12 %)
Neither disagree nor agree	2 (6 %)	5 (15 %)	6 (18 %)	10 (30 %)	0	3 (9 %)	3 (9 %)	10 (30 %)
Agree	2 (6 %)	0	3 (9 %)	1 (3 %)	2 (6 %)	2 (6 %)	1 (3 %)	4 (12 %)
Strongly agree	0	1 (3 %)	8 (24 %)	2 (6 %)	4 (12 %)	3 (9 %)	1 (3 %)	8 (24 %)
**q7.5**	**GAS**	**IMM**	**RHE**	**< 10 years**	**Between 10 and 20 years**	**> 20 years**	**Office**	**Hospital**
Strongly disagree	0	0	2 (6 %)	0	0	2 (6 %)	1 (3 %)	1 (3 %)
Disagree	0	0	1 (3 %)	0	1 (3 %)	0	1 (3 %)	0
Neither disagree nor agree	2 (6 %)	2 (6 %)	3 (9 %)	4 (12 %)	1 (3 %)	2 (6 %)	1 (3 %)	6 (18 %)
Agree	2 (6 %)	5 (15 %)	3 (9 %)	7 (21 %)	3 (9 %)	0	0	10 (30 %)
Strongly agree	0	1 (3 %)	12 (36 %)	5 (15 %)	2 (6 %)	6 (18 %)	4 (12 %)	9 (27 %)
**q7.6**	**GAS**	**IMM**	**RHE**	**< 10 years**	**Between 10 and 20 years**	**> 20 years**	**Office**	**Hospital**
Strongly disagree	0	0	0	0	0	0	0	0
Disagree	0	1 (3 %)	0	1 (3 %)	0	0	0	1 (3 %)
Neither disagree nor agree	1 (3 %)	3 (9 %)	1 (3 %)	4 (12 %)	0	1 (3 %)	0	5 (15 %)
Agree	3 (9 %)	3 (9 %)	13 (39 %)	9 (27 %)	5 (15 %)	5 (15 %)	4 (12 %)	15 (45 %)
Strongly agree	0	1 (3 %)	7 (21 %)	2 (6 %)	2 (6 %)	4 (12 %)	3 (9 %)	5 (15 %)
**q7.7**	**GAS**	**IMM**	**RHE**	**< 10 years**	**Between 10 and 20 years**	**> 20 years**	**Office**	**Hospital**
Strongly disagree	0	0	0	0	0	0	0	0
Disagree	1 (3 %)	1 (3 %)	0	2 (6 %)	0	0	0	2 (6 %)
Neither disagree nor agree	1 (3 %)	2 (6 %)	2 (6 %)	3 (9 %)	0	2 (6 %)	0	5 (15 %)
Agree	2 (6 %)	4 (12 %)	13 (39 %)	9 (27 %)	5 (15 %)	5 (15 %)	4 (12 %)	15 (45 %)
Strongly agree	0	1 (3 %)	6 (18 %)	2 (6 %)	2 (6 %)	3 (9 %)	3 (9 %)	4 (12 %)
**q7.8**	**GAS**	**IMM**	**RHE**	**< 10 years**	**Between 10 and 20 years**	**> 20 years**	**Office**	**Hospital**
Strongly disagree	0	0	1 (3 %)	0	0	1 (3 %)	0	1 (3 %)
Disagree	0	0	2 (6 %)	0	2 (6 %)	0	1 (3 %)	1 (3 %)
Neither disagree nor agree	1 (3 %)	1 (3 %)	0	1 (3 %)	1 (3 %)	0	0	2 (6 %)
Agree	3 (9 %)	6 (18 %)	5 (15 %)	9 (27 %)	1 (3 %)	4 (12 %)	3 (9 %)	11 (33 %)
Strongly agree	0	1 (3 %)	13 (39 %)	6 (18 %)	3 (9 %)	5 (15 %)	3 (9 %)	11 (33 %)

GAS = gastroenterology, IMM = immunoallergology, RHE = rheumatology

Supplementary Table S40. Ordinal data for questions q7.1-13, by specialty, years of cumulative experience and place of practice (3/3). Percentages have been rounded and are for information purposes only. Totals do not necessarily add up to 100%.

q7.9	GAS	IMM	RHE	< 10 years	Between 10 and 20 years	> 20 years	Office	Hospital
Strongly disagree	2 (6 %)	1 (3 %)	3 (9 %)	1 (3 %)	0	0	0	1 (3 %)
Disagree	0	3 (9 %)	4 (12 %)	1 (3 %)	1 (3 %)	0	0	2 (6 %)
Neither disagree nor agree	2 (6 %)	2 (6 %)	5 (15 %)	4 (12 %)	2 (6 %)	1 (3 %)	1 (3 %)	6 (18 %)
Agree	0	2 (6 %)	5 (15 %)	8 (24 %)	2 (6 %)	4 (12 %)	4 (12 %)	10 (30 %)
Strongly agree	0	0	4 (12 %)	2 (6 %)	2 (6 %)	5 (15 %)	2 (6 %)	7 (21 %)
**q7.10**	**GAS**	**IMM**	**RHE**	**< 10 years**	**Between 10 and 20 years**	**> 20 years**	**Office**	**Hospital**
Strongly disagree	0	1 (3 %)	2 (6 %)	0	2 (6 %)	1 (3 %)	1 (3 %)	2 (6 %)
Disagree	2 (6 %)	4 (12 %)	8 (24 %)	10 (30 %)	2 (6 %)	2 (6 %)	2 (6 %)	12 (36 %)
Neither disagree nor agree	1 (3 %)	2 (6 %)	7 (21 %)	3 (9 %)	1 (3 %)	6 (18 %)	2 (6 %)	8 (24 %)
Agree	1 (3 %)	1 (3 %)	3 (9 %)	3 (9 %)	2 (6 %)	0	1 (3 %)	4 (12 %)
Strongly agree	0	0	1 (3 %)	0	0	1 (3 %)	1 (3 %)	0
**q7.11**	**GAS**	**IMM**	**RHE**	**< 10 years**	**Between 10 and 20 years**	**> 20 years**	**Office**	**Hospital**
Strongly disagree	0	0	1 (3 %)	0	0	1 (3 %)	0	1 (3 %)
Disagree	1 (3 %)	2 (6 %)	3 (9 %)	2 (6 %)	2 (6 %)	2 (6 %)	1 (3 %)	5 (15 %)
Neither disagree nor agree	0	3 (9 %)	6 (18 %)	5 (15 %)	2 (6 %)	2 (6 %)	2 (6 %)	7 (21 %)
Agree	3 (9 %)	2 (6 %)	10 (30 %)	8 (24 %)	3 (9 %)	4 (12 %)	3 (9 %)	12 (36 %)
Strongly agree	0	1 (3 %)	1 (3 %)	1 (3 %)	0	1 (3 %)	1 (3 %)	1 (3 %)
**q7.12**	**GAS**	**IMM**	**RHE**	**< 10 years**	**Between 10 and 20 years**	**> 20 years**	**Office**	**Hospital**
Strongly disagree	0	0	0	0	0	0	0	0
Disagree	0	0	0	0	0	0	0	0
Neither disagree nor agree	0	2 (6 %)	1 (3 %)	2 (6 %)	1 (3 %)	0	0	3 (9 %)
Agree	4 (12 %)	5 (15 %)	8 (24 %)	8 (24 %)	4 (12 %)	5 (15 %)	4 (12 %)	13 (39 %)
Strongly agree	0	1 (3 %)	12 (36 %)	6 (18 %)	2 (6 %)	5 (15 %)	3 (9 %)	10 (30 %)
**q7.13**	**GAS**	**IMM**	**RHE**	**< 10 years**	**Between 10 and 20 years**	**> 20 years**	**Office**	**Hospital**
Strongly disagree	0	0	1 (3 %)	0	0	1 (3 %)	1 (3 %)	0
Disagree	2 (6 %)	1 (3 %)	2 (6 %)	1 (3 %)	3 (9 %)	1 (3 %)	1 (3 %)	4 (12 %)
Neither disagree nor agree	1 (3 %)	4 (12 %)	5 (15 %)	8 (24 %)	1 (3 %)	1 (3 %)	1 (3 %)	9 (27 %)
Agree	1 (3 %)	3 (9 %)	9 (27 %)	6 (18 %)	2 (6 %)	5 (15 %)	3 (9 %)	10 (30 %)
Strongly agree	0	0	4 (12 %)	1 (3 %)	1 (3 %)	2 (6 %)	1 (3 %)	3 (9 %)

GAS = gastroenterology, IMM = immunoallergology, RHE = rheumatology
